# Hodgkin’s lymphoma presenting as thoracic telangiectasias: A case report

**DOI:** 10.1177/2050313X241289592

**Published:** 2024-10-07

**Authors:** Mandana Fadaei Kermani, Julie Desrochers

**Affiliations:** 1 Université de Montréal Faculté de Médecine, Montreal, QC, Canada; 2 Hôpital Charles-Le Moyne, Greenfield Park, QC, Canada; 3 Faculty of Medicine and Health Sciences, University of Sherbrooke, Sherbrooke, QC, Canada

**Keywords:** Hodgkin’s lymphoma, dermatology, oncology

## Abstract

The rarity of cutaneous manifestations in Hodgkin’s lymphoma (HL) complicates diagnosis in affected patients. We present the case of a 31-year-old male presenting with a patch of thoracic telangiectasia that led to a dx of HL.

## Introduction

Cutaneous manifestations of Hodgkin’s lymphoma (HL) are only seen in 0.5%–3.5% of cases, making it rather rare and are most often present in cases of advanced HL.^1,2^ This unique case highlights the importance of a comprehensive diagnostic approach in young individuals presenting with unusual vascular manifestations, as it may lead to the early detection of underlying malignancies.

## Case report

**Figure 1.**
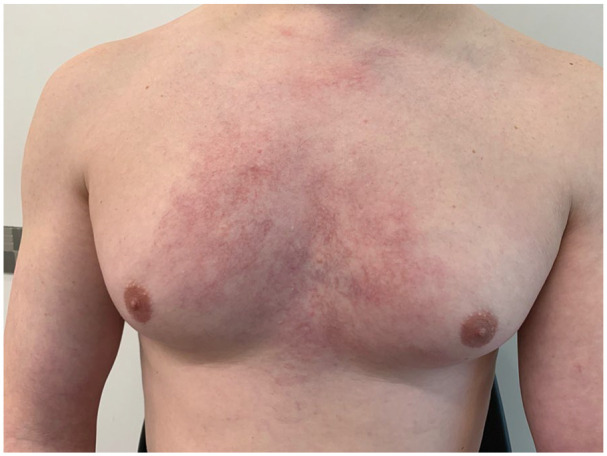
Pretreatment photograph showing the lesion.

**Figure 2.**
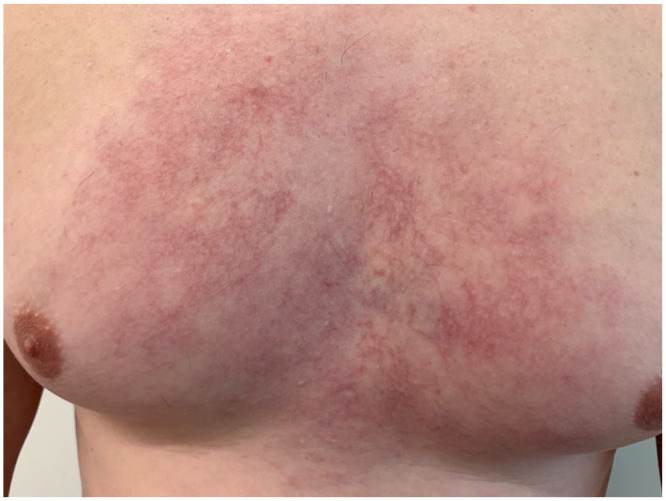
Close-up photograph of the lesion upon initial presentation.

We report a 31-year-old male who came to our clinic complaining of telangiectasias evolving over the past 6 months. Upon presentation, he was systemically in good health. Physical examination revealed red patches composed with livedoid telangiectasia with large irregular mesh over the thorax as seen in Figures 1 and 2. He complained of mild itching, but without chest pain or discomfort. The patient denied using a hot patch over the lesion, aiming to exclude the diagnosis of erythema ab igne. There were no nodules or suspicious lesions otherwise. Denying any dyspnea, coughing, or visual changes, the patient exhibited no signs of venous distension in the neck or chest wall, facial or upper extremity edema, or cyanosis upon physical examination, Making vena cava syndrome less likely.

Our first investigation was a chest X-ray, that showed suprahilar enlargement of the left upper mediastinum with dense and more prominent appearance of the left hilum. A thoracic CT scan with contrast was then performed and revealed an anterior mediastinal suspicious mass with parasternal extension especially around the left manubrium and presternal soft tissue extension, associated right axillary adenomegaly and left supraclavicular adenomegaly. An fluorodeoxyglucose – positron emission tomography (FDG PET-CT) scan reveal that the mass and lymphadenopathy had intense and heterogeneous hypermetabolism which confirms the highly probable neoplastic etiology. Moreover, it showed bony, pericardial, and muscular invasion by contiguity.

**Figure 3.**
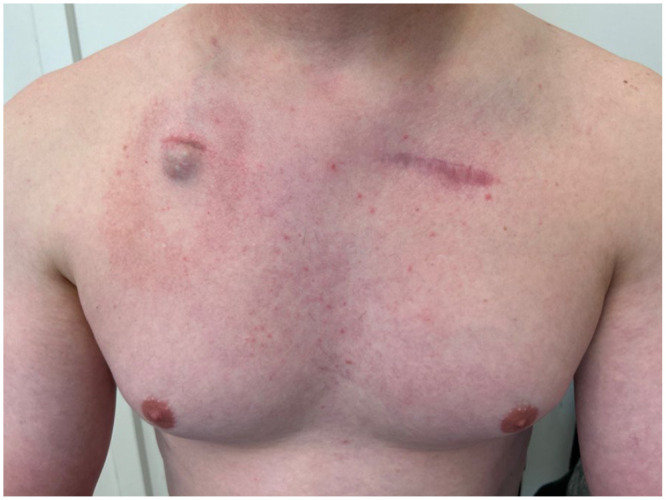
Image showing the evolution of the lesion posttreatment.

The diagnosis of HL stage IIBE was retained. The patient was treated with 12 cycles of adriamycin, bleomycin sulfate, vinblastine sulfate and dacarbazine (ABVD) chemotherapy. At the end of his course of treatment, the FDG PET-CT scan showed a complete metabolic response with decreased metabolism of lymphomatous involvement in the anterior mediastinum and right anterior chest wall (Deauville score 3) with no new hypermetabolic focus suspicious for lymphomatous invasion. At his follow-up appointment, there was a complete disappearance of the vascular spot on the thorax as seen in Figure 3. His sole dermatological condition was steroid acne arising as a secondary effect of chemotherapy.

## Discussion

The incidence of HL is around 0.98 per 100,000.^3^ HL represents 0.4% of all newly reported cancer cases.^4^ Its incidence has a bimodal peak, from 25 to 30 years of age, and from 75 to 80 years of age. The etiology and pathogenesis are not known. However, a few risk factors have been identified such as genetic predisposition, infections with Epstein–Barr virus or HIV, and immune suppression.^5,6^

Cutaneous manifestations from HL are unusual and nonspecific. They include pruritus, hyperpigmentation, urticaria, erythroderma, or acquired ichthyosis. However, these cutaneous presentations are thought to be paraneoplastic syndromes rather than caused by direct tumor infiltration. Cutaneous lesions by direct tumor infiltration are much rarer, at a reported frequency of 0.5%–3.4%.^5,7^ These lesions present more commonly as plaques, nodules, papules, or ulcers and typically occur in advanced stages.^8^ The pathogenesis for the skin involvement in HL is not known, but three mechanisms have been proposed: (1) retrograde lymphatic spread from tumor-involved lymph nodes, (2) direct extension by tumor cells in underlying lymph nodes, or (3) hematogenous dissemination of the tumor.^7,9^ Our hypothesis for the telangiectasias included possible venous congestion secondary to the mass and lymphadenopathy or even direct infiltration of the dermal vessels by the lymphoma. Unfortunately, the first investigation was a chest X-ray, so no biopsy was done. It could have been interesting to have the histology report to better understand the exact pathogenesis of our case.

There is no standard treatment for cutaneous HL. Treatment options include systemic chemotherapy with or without radiotherapy, local treatment with topical agents, or local radiotherapy alone.^9^ In our case, the patient has been evaluated by the oncology team and treated with ABVD chemotherapy given the classification according to Ann Arbor staging.

This case underscores the importance of considering malignancies, such as lymphomas, in the differential diagnosis of young individuals presenting with thoracic telangiectasia. Although the association between telangiectasia and HL is rare, a thorough diagnostic workup is crucial when facing an atypical presentation to identify and promptly manage underlying malignancies in such cases.

## Conclusion

The concurrent presentation of thoracic telangiectasia and HL in a young adult emphasizes the need for a multidisciplinary approach to diagnosis and management. Early recognition and appropriate treatment are essential for achieving favorable outcomes in these complex cases.
